# Oral semaglutide improves body composition and preserves lean mass in patients with type 2 diabetes: a 26-week prospective real-life study

**DOI:** 10.3389/fendo.2023.1240263

**Published:** 2023-09-13

**Authors:** Sara Volpe, Giuseppe Lisco, Margherita Fanelli, Davide Racaniello, Valentina Colaianni, Valentina Lavarra, Domenico Triggiani, Lucilla Crudele, Vincenzo Triggiani, Carlo Sabbà, Giovanni De Pergola, Giuseppina Piazzolla

**Affiliations:** ^1^ Interdisciplinary Department of Medicine, School of Medicine, University of Bari “A. Moro”, Bari, Italy; ^2^ Unit of Geriatrics and Internal Medicine, National Institute of Gastroenterology—IRCCS “Saverio de Bellis”, Bari, Italy

**Keywords:** oral semaglutide, body composition, fat mass, visceral adipose tissue, fat free mass, skeletal muscle mass, type 2 diabetes, GLP-1RA

## Abstract

**Background:**

Oral semaglutide is the first glucagon-like peptide-1 receptor agonist (GLP-1RA) designed for oral administration; it offers a promising opportunity to facilitate an early approach to Type 2 Diabetes (T2D). The study aimed to evaluate, in a real-life setting, the effects of oral semaglutide on the body composition of patients with T2D after 26 weeks of therapy.

**Methods:**

Thirty-two patients with T2D were evaluated at baseline (T0) and after three (T3) and six (T6) months of therapy with oral semaglutide. At each time point, body composition was assessed using a phase sensitive bioimpedance analyzer. Clinical, anthropometric and laboratory parameters, and the main biometric surrogates of liver steatosis and fibrosis, were also analyzed and compared.

**Results:**

A significant and early reduction in anthropometric and glucometabolic parameters, alanine aminotransferase, Fatty Liver Index, and Fat Mass was observed. Visceral Adipose Tissue (VAT) decreased, while Fat Free Mass and Skeletal Muscle Mass (SMM) were preserved during therapy, resulting in a beneficial increase in the SMM/VAT ratio. Finally, an overall improvement in body fluid distribution was observed.

**Conclusion:**

Our real-world data confirm the clinical efficacy of oral semaglutide and highlight its ability to improve the nutritional status of patients with T2D.

## 1 Introduction

The prevalence of type 2 diabetes (T2D) is rising globally, along with other chronic pathophysiologically and epidemiologically related diseases, including overweight and obesity, arterial hypertension, liver steatosis, and dyslipidemia, all conditions fostered by a sedentary lifestyle and diets high in hydrogenated fats and high glycemic load (1). Concordantly, the importance of a multifactorial approach in T2D has been emphasized by some authors (2, 3). The management of T2D has changed dramatically in the last decade due to the availability of new molecules with proven glycemic and extra-glycemic effects, targeting some specifically diabetes-related chronic comorbidities such as obesity and liver steatosis (4, 5). As an example, glucagon-like peptide-1 receptor agonists (GLP-1RAs) are potent antidiabetic drugs with a favorable safety profile, also allowing a significant weight loss and protective cardiovascular (CV) effects (6, 7)

The use of peptides as oral therapeutic drugs is a long-standing challenge that poses significant technical, physiological and interindividual variability-related obstacles. Oral semaglutide is the first GLP1-RA available for oral administration. For the first time, a peptide-based molecule has been designed to undergo gastric absorption, avoiding the pH-dependent degradation and demolition operated by peptic enzymes (8). More precisely, oral semaglutide has been developed in coformulation with sodium N-[8-(2-hydroxybenzoyl) amino] caprylate (SNAC), which acts as an absorption enhancer. SNAC is a hydrophobic molecule that binds to semaglutide, inducing an increased transcellular lipophilicity and enhanced mucosal absorption, without the need for a protective enteric coating (9). The activity of SNAC is transient and reversible, so it separates from the medication soon after it enters the bloodstream. Oral semaglutide is available in three doses: 3, 7, and 14 mg. It needs to be carefully titrated to avoid gastrointestinal discomfort, with a starting dose of 3 mg/day for the first 30 days, followed by 7 mg/day and then, if necessary, 14 mg. During the Peptide Innovation for Early Diabetes Treatment (PIONEER) program (10–15), oral semaglutide (14 mg) reduced HbA1c levels by 1.0-1.4%, significantly more than sitagliptin or empagliflozin did, and to a similar extent to liraglutide after 26 weeks. It also reduced body weight (BW) more than sitagliptin and liraglutide, and to a similar extent to empagliflozin. Lastly, it showed superiority in terms of both HbA1c, and BW reduction compared to dulaglutide (16). Although studies directly comparing the efficacy and safety of oral and subcutaneous semaglutide have never been conducted, a recently published systematic review and network meta-analysis showed that oral semaglutide was slightly less effective than subcutaneous semaglutide in improving glucose control and BW, with a comparable safety profile (17).

Weight loss is essential to improve the management of T2D and related comorbidities and complications, especially fat mass loss while preserving lean mass. In fact, weight loss due to a major decline in lean body mass is normally associated with a decline in skeletal muscle mass and strength, resulting in an increased risk of sarcopenia, impaired glucose sensitivity, and poor glucose control (18). Coexisting obesity and sarcopenia, also called sarcopenic obesity, is one of the leading causes of long-term worsening of glucose control, and should be considered another crucial therapeutic target, especially in elderly patients with T2D (19, 20).

The liver plays a central role in the regulation of lipid metabolism as it is the foremost site of lipid uptake, synthesis, oxidation, and distribution to peripheral tissues. Non Alcoholic Fatty Liver Disease (NAFLD) consists of excessive lipid accumulation in the liver, lipotoxicity, inflammation, and potential progression to Non Alcoholic SteatoHepatitis (NASH), liver fibrosis, and hepatocellular carcinoma (21). Evidence suggests a bidirectional relationship between the progression of NAFLD and T2D (22). An up-to-date approach to T2D should include appropriate assessment of body composition and biomarkers of NAFLD, both for clinical and research purposes, due to the metabolic role of new antihyperglycemic drugs, whose effect on weight, body composition, and metabolic parameters is now well established and documented (23–27).

In this context, early improvement in body composition, assessed by segmental multifrequency bioelectrical impedance analysis (SMF-BIA) (27), and in biochemical and ultrasound (US) characteristics of Non-Alcoholic Fatty Liver Disease (NAFLD) (28) has recently been found in T2D patients treated with once-weekly semaglutide for 26 and 52 weeks. On this basis, the aim of this study was to evaluate the effects of oral semaglutide on body composition and surrogate markers of NAFLD in a cohort of patients with T2D followed up for 26 consecutive weeks in a real-life setting.

## 2 Methods

### 2.1 Study design and institution

This is a 26-weeks, open-label, real-life prospective study conducted at the Outpatient Section for Metabolic Disorders of the Department of Internal Medicine, University of Bari. The study protocol was approved by the Ethics Committee of the Bari Polyclinic University Hospital (protocol number 6468 version 2, on 09/14/2020).

### 2.2 Study protocol

In total, 130 patients with T2D attending our Clinic were screened for eligibility from December 01, 2021, to May 31, 2022. At initial observation, all patients were given general advice about the importance of improving lifestyle, with no specific prescription in terms of daily physical exercise. To be included in the study, patients had to have an established diagnosis of T2D; age ≥18 years; estimated glomerular filtration rate (eGFR) >15 mL/min/1.73 m^2^, and had to be eligible for GLP1 prescription according to current recommendations and guidelines, namely: i) to achieve adequate glucose control, i.e., glycated hemoglobin (HbA1c) <7% or individualized goals (HbA1c <6.5% for most); ii) regardless of baseline HbA1c, to manage cases with established CV disease or high cardiovascular risk (CVR), i.e. the presence of documented atherosclerotic vascular damage, or damage in a target organ or at least three CVR factors (among age >50 years, hypertension, dyslipidemia, obesity, cigarette smoking); iii) when there was a need to minimize weight gain or promote weight loss.

Exclusion criteria included other forms of diabetes mellitus, pregnancy or lactation, contraindications to GLP-1RAs, previous or ongoing treatment with pioglitazone and/or Sodium-glucose cotransporter-2 inhibitors (SGLT2i) and/or GLP-1RAs, inadequate ability to comply with follow-up or provide informed consent, implantable electronic devices (cardioverter defibrillators or pacemakers) contraindicating body composition assessment by segmental multifrequency bioelectrical impedance analysis (SMF-BIA- Seca mBCA 525), as indicated by the manufacturer (Seca GmbH & Co., KG, Hamburg, Germany), the use of oral contraceptives or corticosteroids, infection by viral hepatitis B and C, and excessive ethanol consumption (more than 30 grams/day in men and 20 grams/day in women).

**Figure 1:**
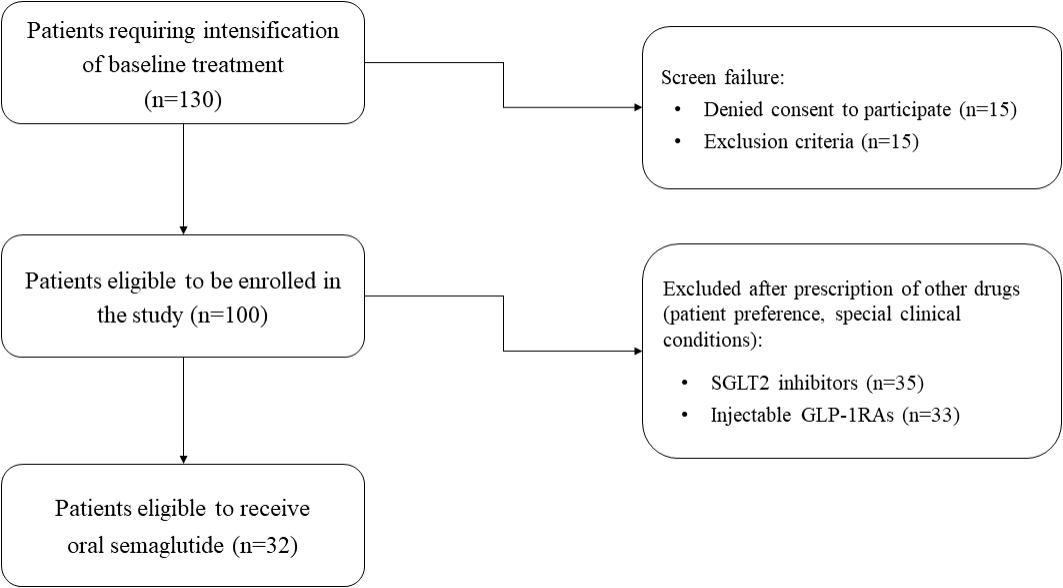
The flow chart illustrates the process of selecting patients enrolled in the study.

As shown in detail in **Figure 1**, the study finally included 32 patients (mean age 66.3 ± 8.5 years; men 43.3%), who agreed to start treatment with oral semaglutide and, after being fully informed about the purposes of the study, gave written consent for participation. Oral semaglutide was administered in accordance with general recommendations. As per good clinical practice, a daily dose of 3 mg was prescribed for 30 days at the first visit and increased to 7 mg per day starting from the second month. No patients needed a further increase to 14 mg/day during the follow-up. All participants were on metformin at the maximum tolerated dose according to clinical and laboratory parameters (mean dose = 1500 mg), and none were taking other antihyperglycemic drugs including insulin analogues. All medications being taken by enrolled patients at baseline are listed in **Table 1**.

**Table 1:** List of drugs taken by enrolled patients at baseline.

Medications	Number of patients
Metformin	32/32 (100%)
Antihypertensive agents - angiotensin receptor blockers - β-blockers - calcium-channel antagonists - diuretics - angiotensin-converting enzyme inhibitors	26/32 (81%) 17/26 (65.4%) 9/26 (34.6%) 7/26 (26.9%) 8/26 (30.8%) 4/26 (15.4%)
Anti-hypercholesterolemic agents - Statins - Statin + Ezetimibe	28/32 (87.5%) 22/28 (78.6%) 6/28 (21.4%)
Polyunsaturated fatty acids	1/32 (3.1%)

The study period lasted 26 consecutive weeks. Visits were scheduled at baseline (T0), after three (T3) and six months of therapy (T6), and a complete history and physical examination were performed each time. Anthropometrical and clinical parameters were systolic and diastolic blood pressure, heart rate, BW, waist circumference (WC), and body mass index (BMI). Laboratory tests included: total blood count, fasting plasma glucose (FPG), glycated hemoglobin (HbA1c), serum creatinine and eGFR, fasting plasma insulin, c-peptide, liver enzymes (aspartate aminotransferase, AST; alanine aminotransferase, ALT; γ-glutamyl transferase, γGT), total cholesterol, Low-Density Lipoprotein (LDL)-cholesterol, High-Density Lipoprotein (HDL)-cholesterol, triglycerides, and serum pancreatic enzymes (pancreatic isoamylase and lipase).

We used the HOMA-IR (HOmeostasis Model Assessment of Insulin Resistance) index as an indirect measure of systemic insulin resistance, and it was calculated as (fasting insulin × fasting glucose)/405 (normal range 0.23–2.5).

The risk of liver fibrosis and cirrhosis was estimated by the AST to Platelet Ratio Index (APRI) score, calculated according to the following formula: [(AST/upper normal value] × 100]/number of platelets. Values above 0.5 or 1 were considered indicative of an increased risk of liver fibrosis or cirrhosis, respectively (29).

The Hepatic Steatosis Index (HSI), used to estimate the risk of liver steatosis, was calculated according to the following formula: [8 × (AST/ALT) + BMI + 2 + 2 if female]. A value above 36 was suggestive of NAFLD, and a value lower than 30 should exclude the disease (30).

The Fatty Liver Index (FLI) was calculated according to the formula: FLI = (e0.953 × ln (triglycerides) + 0.139 × BMI + 0.718 × ln (γGT) + 0.053 × WC − 15.745)/(1 + e0.953 × ln (triglycerides) + 0.139 × BMI + 0.718 × ln (γGT) + 0.053 × WC − 15.745) × 100. An FLI value below 30 (negative likelihood ratio up to 0.2) suggests excluding the presence of liver steatosis, whereas an FLI equal to or higher than 60 (positive likelihood ratio starting from 4.3) was considered indicative of liver steatosis (31).

The FIB4 (Fibrosis-4) index for liver fibrosis was calculated as follows: FIB-4 = (AST x Age)/(Platelet count x √(ALT)). A cut off <1.45 has a negative predictive value of 90% for ruling out extensive fibrosis. A cutoff value > 3.25 has a positive predictive value of 65% for the diagnosis of extensive fibrosis (32).

### 2.3 Assessment of body composition

The assessment of body composition was performed by a phase-sensitive octopolar SMF-BIA operating with Seca mBCA 525 (Seca GmbH & Co., KG, Hamburg, Germany) and Seca Analytics 115 software. The method is completely standardized. Briefly, each patient, fasting for 8 h and resting for at least 8 h, was placed in supine position with each leg at a 45° angle and each arm at a 30° angle from the trunk.

Eight electrodes (Kendall, H59P, Covidien IIc, Mansfield, MA, USA) were placed on each side, with the proximal edge of the first electrode connected by an imaginary line to the styloid process of the ulna, and the distal edge of the finger electrode on an imaginary line through the center of the metacarpophalangeal joints of the index and middle fingers. The distal edge of the toe electrode was placed along an imaginary line crossing the center of the metatarsophalangeal joints of the second and third toes. The proximal edge of the ankle joint electrode was connected along a line crossing the highest points of the outer and inner ankle bones.

The body impedance was measured using an alternating current at 100 µA with frequencies between 1 and 500 kHz, while the raw data of Resistance (R) and Reactance (Xc) were obtained at 50 Khz, and used to automatically calculate the Phase Angle by the BIA device, with the formula Phase Angle = Reactance (xc)/Resistance (R)* (180/π).

Seca Analytics 115 software, in accordance with previous validation studies, automatically calculated the following data: Skeletal Muscle Mass (SMM), Skeletal Muscle Index (SMI) and Visceral Adipose Tissue (VAT) (33), Total Body Water (TBW), Extracellular Water (ECW), Fat-Free Mass (FFM) and Fat Mass (FM) (34).

Fat-Free Mass Index (FFMI) and Fat Mass Index (FMI) were expressed as the ratio Fat-Free Mass (Kilograms) to Height Squared (mt2) and Fat Mass (Kilograms) to Height Squared (mt2), respectively.

### 2.4 Study outcomes

The primary outcome was the estimation of mean changes in body composition, paying particular attention to Fat Mass (FM) and Fat Free Mass (FFM), from T0 to T3 and T6.

Secondary outcomes were the mean changes in anthropometric parameters, such as body weight, BMI, and waist circumference, glucose control (FPG, HbA1c, insulin resistance), and validated clinical scores predicting the risk of NAFLD (HSI, FLI, APRI, and FIB4) over the entire observation period (T0, T3, and T6). A body weight loss of at least 5% from T0 to 3 and 6 months was considered relevant. Patients who had lost at least 5% of baseline body weight were defined as Responders (R), while Non-Responders (NR) were those who did not.

## 3 Statistical analysis

To assess whether the available sample was sufficient to obtain reliable results, the first step was to evaluate the power analysis, using G*power, for the variable Fat Mass, which is one of the primary outcomes. Assuming an explained variability of at least 15% (η^2^), setting α=0.05, an effect size of 0.4 corresponding to a test power of 0.9 was estimated, based on a real sample of 30 patients (35).

Patient characteristics at baseline were expressed as mean, standard deviation, median, range, frequency, and percentage, as appropriate. Changes over time (T0, T3 and T6) for each variable were analyzed with repeated-measures Mixed Models, and changes in the means were estimated by the least-squares method; pre-planned contrasts between times were inserted in the model. To assess potential predictive factors, baseline characteristics were compared between Responder and Non-Responder patients by unpaired Student t test. Statistical analysis was performed with SAS 9.4 software (SAS Institute Inc., Cary, NC, USA), and graphs were made with Microsoft excel for Mac (vers.16.16.27, ©Microsoft 2018).

## 4 Results

### 4.1 Baseline characteristics of the study population

The baseline characteristics of patients are shown in **Table 2**. The patients were mostly women (56.7%; male-to-female ratio 0.76:1). The median duration of T2D was 7.5 years, ranging from 0 (new diagnosis) to 35 years. A high median value ​​of BMI and waist circumference suggested that the study population had a typical dysmetabolic phenotype, even if patients with normal or lower than normal BMI (up to 17.9 kg/m^2^) were also included. More specifically, normal-weight patients (BMI < 25 kg/m^2^) accounted for 12.5% of the study population, while overweight (25 < BMI < 30 kg/m^2^) and first-class obesity (30 < BMI < 35 kg/m^2^) were found in 53.1% and 34.4% of patients, respectively. Insulin resistance (HOMA-IR) and glucose control (HbA1c) showed a wide range of variability. Renal function, lipid profile, liver enzymes and surrogate of liver fibrosis markers (APRI and FIB4) were normal on average while the mean and median values ​​of the main laboratory surrogates of hepatic steatosis (i.e., HSI and FLI) were elevated (**Table 2**). Based on the individual CVR, 5 patients did not achieve adequate LDL-cholesterol control, and the anti-hypercholesterolemic therapy was therefore prescribed, or increased, at T0.

**Table 2:** Baseline characteristics of the study population (n = 32).

Baseline Characteristics	Mean (S.D.)	Median (Min; Max)
Age (years)	66.3 (8.5)	67.5 (50; 80)
Diabetes duration (years)	8.7 (7.6)	7.5 (0; 35)
Body weight (kg)	75.3 (10.8)	73.0 (56; 98)
Body mass index (kg/m^2^)	28.2 (3.3)	28.5 (17.9; 33.6)
Waist circumference (cm)	102.1 (7.9)	104.5 (77; 115)
Fasting glycemia (mg/dL)	119.7 (23.3)	113 (86; 186)
Glycated hemoglobin (mmol/mol)	46.5 (6.0)	44.5 (37; 61)
Fasting serum C-peptide (ng/mL)	2.9 (1.6)	2.5 (0.6; 8.3)
Fasting serum insulin (mmIU/L)	14.8 (16.7)	9.7 (2; 83.9)
HOMA-IR index	4.3 (6.1)	2.4 (0; 31)
Total Cholesterol (mg/dL)	154.5 (28.5)	155 (98; 221)
LDL Cholesterol (mg/dL)	75.6 (25.2)	74 (29; 137)
HDL Cholesterol (mg/dL)	55.6 (14.4)	55 (30; 89)
Triglycerides (mg/dL)	122.6 (68.9)	102 (42; 302)
Serum creatinine (mg/dL)	0.9 (0.3)	0.9 (0.1; 1.6)
eGFR (mL/min/1.73 m^2^)	78.1 (17.7)	77 (31; 108)
AST (IU/L)	22.8 (13.2)	20 (10; 85)
ALT (IU/L)	28.8 (14.5)	25 (10; 65)
γGT (IU/L)	34.4 (21.4)	27 (12; 95)
FLI	60.4 (22.6)	66 (14; 94)
HSI	37.1 (4.1)	37.6 (25.8; 45.4)
APRI	0.1 (0.1)	0.09 (0.04; 0.73)
FIB4	1.6 (1.4)	1.0 (1; 7)

Baseline characteristics are displayed as means, standard deviation, median, minimum, and maximum values. HOMA-IR, HOmeostasis Model Assessment of Insulin Resistance; LDL, Low-Density Lipoprotein; HDL, High-Density Lipoprotein; eGFR, estimated Glomerular Filtration Rate; AST, ASpartate aminoTransferase; ALT, ALanine aminoTransferase; γGT, gamma-Glutamyl Transferase; FLI, Fatty Liver Index; HSI, Hepatic Steatosis Index; APRI, Aspartate aminotransferase to Platelet Ratio Index; FIB4, Index for Liver Fibrosis.

### 4.2 Anthropometric and glucometabolic parameters during the study period

**Figure 2:**
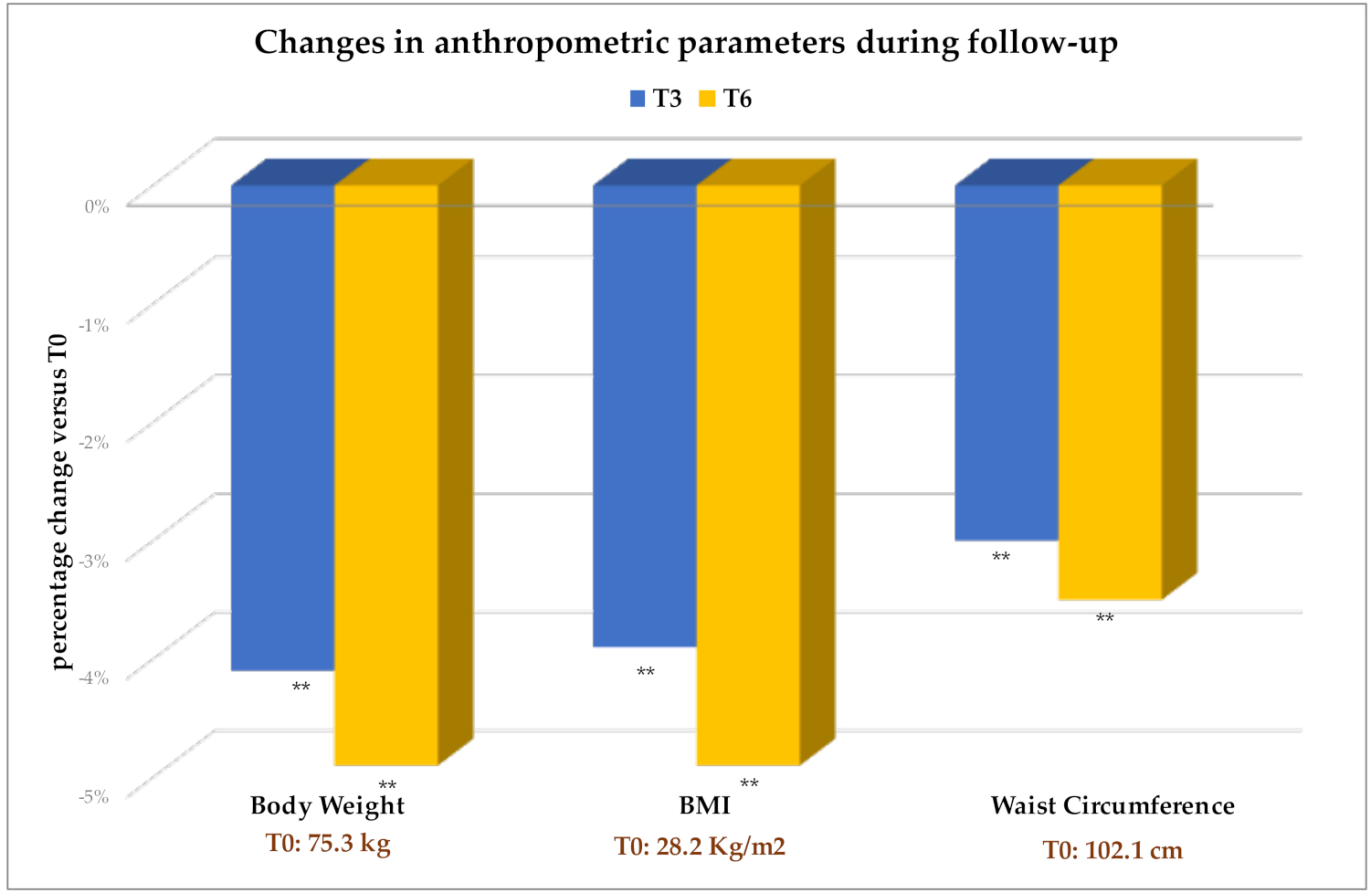
Values are expressed as percent change from baseline (T0). The absolute values of each parameter at T0 are also indicated. Change versus T0: **p<0.001. BMI, Body Mass Index.

Body weight, BMI, and waist circumference were significantly lower after 3 months of therapy and up to T6 (**Figure 2** and **Table 3**). The average weight loss was 3 kg after 3 months, and around 4 kg after 6 months of treatment.

**Table 3:** Estimated means of anthropometric, clinical and laboratory parameters over the study period.

Parameters	Study period		
	T0	T3	T6
Body weight (kg)	75.3 ± 1.9	72.2 ± 1.9**	71.6 ± 1.9**
Body mass index (kg/m2)	28.2 ± 0.6	27.1 ± 0.5**	26.8 ± 0.6**
Waist circumference (cm)	102.1 ± 1.4	99 ± 1.3**	98.5 ± 1.5**
Heart rate (bpm)	74.5 ± 1.8	77.7 ± 2.4	75.7 ± 1.9
Systolic Blood Pressure (mmHg)	128.7 ± 2.3	124.8 ± 2.7	121.9 ± 2.6*
Diastolic Blood Pressure (mmHg)	74.9 ± 1.7	73.5 ± 1.6	75.4 ± 1.7
Fasting glycemia (mg/dL)	119.7 ± 4.1	112.7 ± 4.5	104.5 ± 4.0**#
Glycated hemoglobin (mmol/mol)	46.1 ± 1.0	43.6 ± 1.3**	41.9 ± 1.3**#
eGFR (mL/min/1.73 m2)	78.2 ± 3.1	81.5 ± 4.3	79.3 ± 4.2
Pancreatic isoamylase (IU/L)	35.2 ± 3.6	45.4 ± 5.9	42.4 ± 3.9
Lipase (IU/L)	236.3 ± 36.9	192.4 ± 38.2	243.2 ± 32.7
Total Cholesterol (mg/dL)	154.4 ± 4.8	140.8 ± 3.8**	139.2 ± 3.6**
LDL Cholesterol (mg/dL)	75.7 ± 4.2	67.0 ± 3.2*	65.7 ± 2.7*
HDL Cholesterol (mg/dL)	55.6 ± 2.5	52.5 ± 2.3	51.6 ± 2.2
Triglycerides (mg/dL)	122.6 ± 12	121.2 ± 10.8	111.6 ± 9.0
HOMA-IR index	4.2 ± 1.2	3.3 ± 0.6	3.2 ± 0.7
AST (IU/L)	22.9 ± 2.1	25.1 ± 3.8	23.3 ± 2.6
ALT (IU/L)	28.7 ± 2.5	27.7 ± 4.1	23.7 ± 2.3*
γGT (IU/L)	33.2 ± 3.6	28.1 ± 4.4	37.6 ± 9.5
FLI	61.1 ± 4.2	51.2 ± 4.3**	52.8 ± 4.6**
HSI	37.1 ± 0.7	36.9 ± 0.7	36.9 ± 0.7
APRI	0.13 ± 0.02	0.15 ± 0.04	0.13 ± 0.03
FIB4	1.6 ± 0.2	1.8 ± 0.3	1.8 ± 0.3

Parameters are expressed as mean ± S.E. Estimated changes were analyzed at different times. Change versus T0: *p<0.05; **p<0.001. Change at T6 versus T3: #p<0.05. HOMA-IR: HOMA-IR, HOmeostasis Model Assessment of Insulin Resistance; LDL, Low-Density Lipoprotein; HDL, High-Density Lipoprotein; eGFR, estimated Glomerular Filtration Rate; AST, ASpartate aminoTransferase; ALT, ALanine aminoTransferase; γGT, gamma-Glutamyl Transferase; FLI, Fatty Liver Index; HSI, Hepatic Steatosis Index; APRI, Aspartate aminotransferase to Platelet Ratio Index; FIB4, Index for Liver Fibrosis.

Regarding body weight at T6, 16 patients (50%) were classified as R (7 men and 12 women). Baseline characteristics of R and NR were not significantly different and no predictive factors, such as gender, anthropometric, clinical, and laboratory parameters, were found to explain the heterogeneity in the individual response to oral semaglutide in terms of weight loss (data not shown).

**Figure 3:**
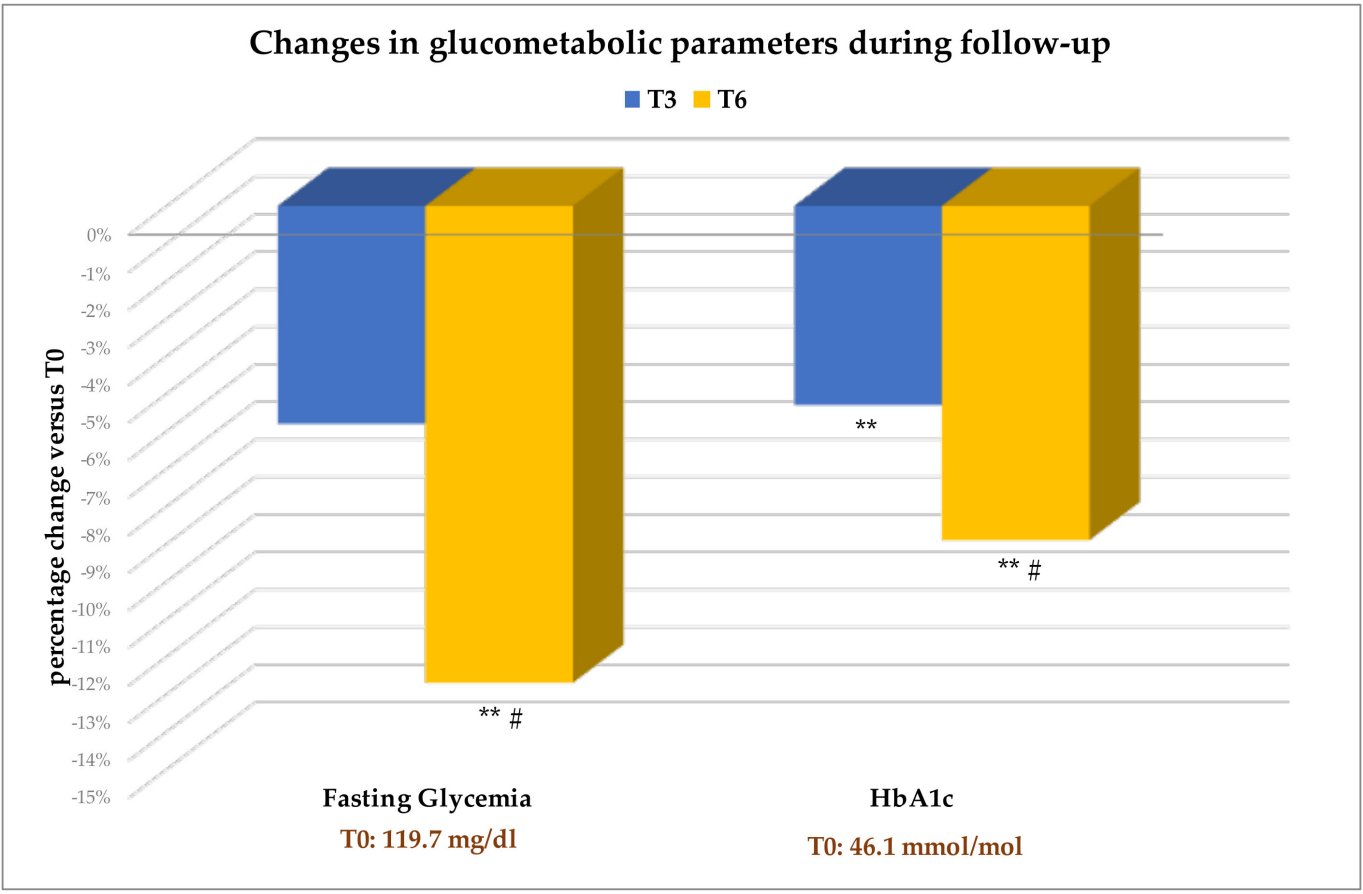
Values are expressed as percent change from baseline (T0). The absolute values of each parameter at T0 are also indicated. Change versus T0: **p<0.001. Change at T6 versus T3: #p<0.05. HbA1c, Glycated hemoglobin.

Patients experienced a significant reduction in fasting glycemia and HbA1c (**Table 3**). As shown in **Figure 3**, improvements in both parameters were already evident at T3 compared with baseline, with further improvements at T6 compared to T3.

### 4.3 Changes in clinical and laboratory parameters during follow-up

Although GLP-1RAs have a mild positive chronotropic effect, heart rate never reached a significant increase during the treatment period with oral semaglutide (**Table 3**). In contrast, a significant decrease in systolic blood pressure occurred at T6 compared with T0 (**Table 3**).

**Figure 4:**
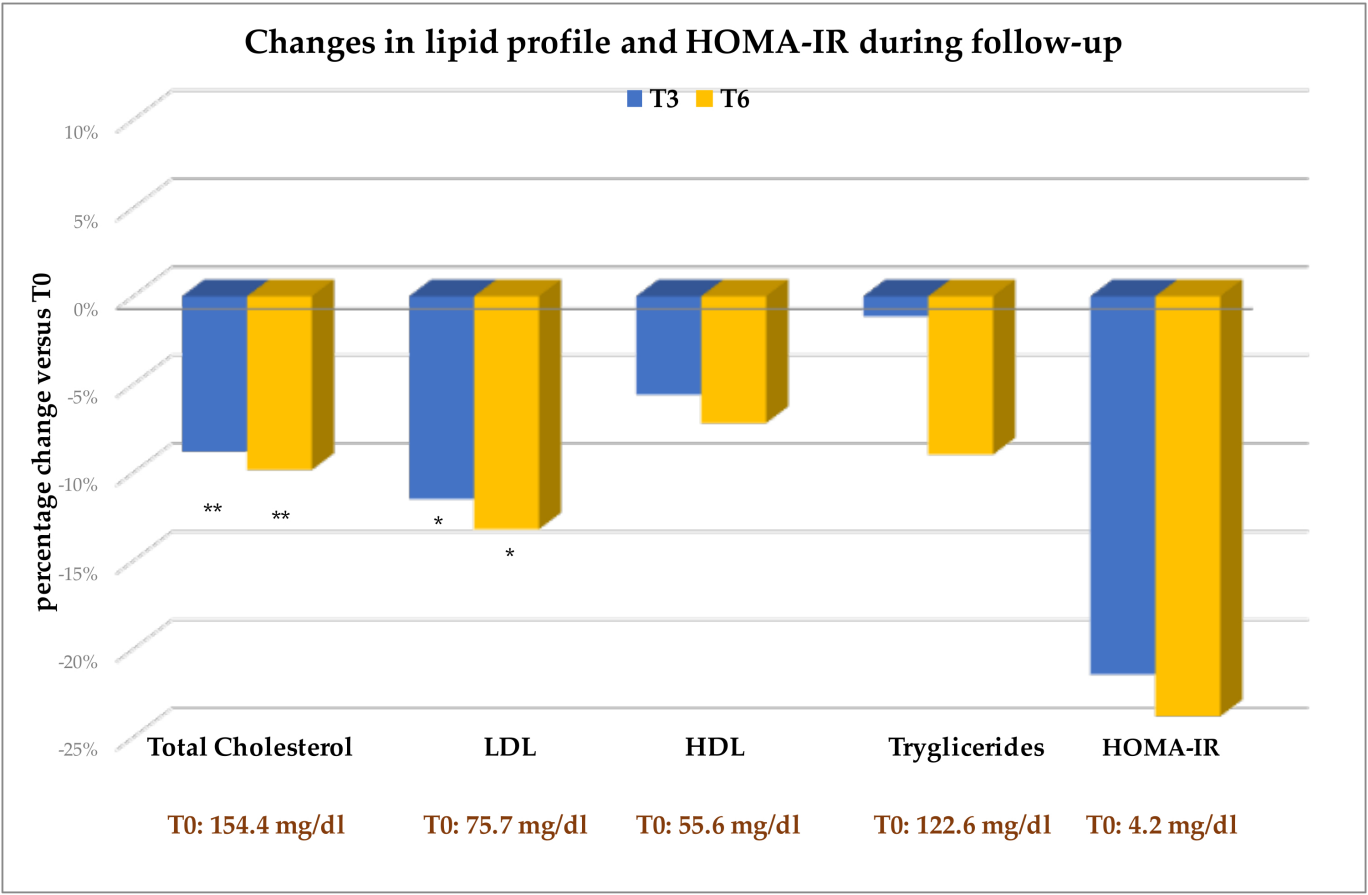
Mean changes, expressed as percentage changes from baseline (T0 = 100%). Change versus T0: *p<0.05; **p<0.001. eGFR, estimated Glomerular Filtration Rate; LDL, Low-Density Lipoprotein; HDL, High-density Lipoprotein; HOMA-IR, HOmeostasis Model Assessment of Insulin Resistance.

Changes in eGFR during therapy were negligible (**Table 3**). As regards pancreatic enzymes, only pancreatic isoamylases increased slightly under oral semaglutide therapy, but this increase was not significant compared with T0 at any time (**Table 3**). Total and LDL cholesterol decreased significantly from T0 to T3 and T6 (**Table 3** and **Figure 4**). Serum triglycerides, HDL cholesterol, and the HOMA-index decreased throughout the follow-up, but these reductions never reached statistical significance (**Table 3** and **Figure 4**).

Changes in serum AST and γGT were negligible during follow-up, while ALT levels were found to be significantly lower than baseline after 6 months of therapy (**Table 3**). FLI, an important laboratory surrogate of fatty liver disease, decreased significantly as early as T3, and this improvement was maintained up to T6 (**Table 3**). As expected, liver fibrosis indices (the APRI and FIB4 scores), which were normal at baseline, did not change significantly as an effect of therapy (see **Table 3**).

### 4.4 Body composition changes during the study period

**Figure 5:**
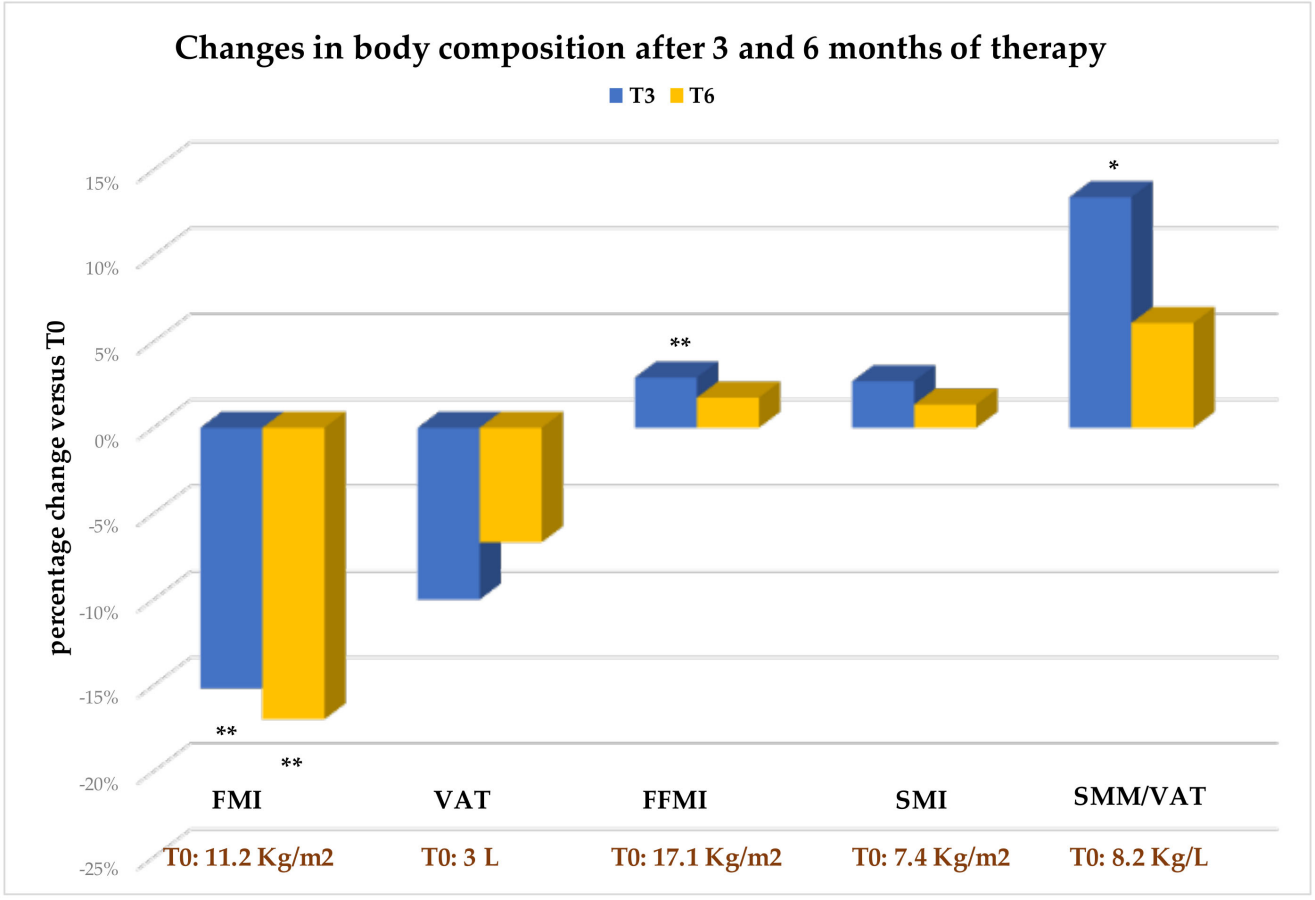
Values are expressed as percent change from baseline (T0). Change versus T0: *p<0.05; **p<0.001. FMI, Fat Mass Index; VAT, Visceral Adipose Tissue; FFMI, Fat Free Mass Index; SMI, Skeletal Muscle Mass Index; SMM, Skeletal Muscle Mass.

Changes in the main bio-impedance parameters describing body composition over the study period are described in **Table 4** and **Figure 5**.

**Table 4:** Estimated means of bioimpedance parameters describing body composition over the study period.

Parameters	Study period		
	T0	T3	T6
Fat Mass (Kg)	29.5 ± 1.2	24.9 ± 1.2**	24.3 ± 1.2**
Fat Mass Index (Kg/m^2^)	11.2 ± 0.5	9.5 ± 0.5**	9.3 ± 0.5**
Fat Mass %	39.3 ± 1.3	34.7 ± 1.5**	34.2 ± 1.5**
Fat-Free Mass (Kg)	45.4 ± 1.4	47.1 ± 1.7**	46.9 ± 1.6**
Fat-Free Mass Index (Kg/m^2^)	17.1 ± 0.3	17.6 ± 0.4**	17.4 ± 0.3
Fat-Free Mass %	60.5 ± 1.3	65.2 ± 1.6**	65.7 ± 1.5**
Skeletal Muscle Mass (SMM; Kg)	20.0 ± 0.8	20.6 ± 1.0	20.3 ± 0.9
Skeletal Muscle Mass Index (Kg/m^2^)	7.4 ± 0.2	7.6 ± 0.2	7.5 ± 0.2
Phase Angle (°)	5.1 ± 0.1	5.2 ± 0.1	5.1 ± 0.1
Visceral Adipose Tissue (VAT; L)	3 ± 0.2	2.7 ± 0.2	2.8 ± 0.2
SMM/VAT (Kg/L)	8.2 ± 1.0	9.3 ± 1.1*	8.7 ± 0.29
Total Body Water (TBW; L)	34.1 ± 1.0	35.2 ± 1.2*	35.0 ± 1.1*
Extracellular Body Water (ECW; L)	16.9 ± 1.1	16.2 ± 0.5	16.2 ± 0.4
ECW/TBW %	46.3 ± 0.5	46.0 ± 0.4	46.6 ± 0.5#

Each parameter is expressed as mean ± S.E. Estimated changes were analyzed at different times. Change versus T0: *p<0.05; **p<0.001. Change at T6 versus T3: #p<0.05. SMM, Skeletal Muscle Mass; VAT, Visceral Adipose Tissue; ECW, Extracellular Body Water; TBW, Total Body Water.

A significant and early reduction in both total Fat Mass (**Table 4**) and Fat Mass Index (FMI, **Table 4** and **Figure 5**) was found. Conversely, the reduction in VAT, although documented at both T3 and T6 compared with T0, did not reach statistical significance (see **Table 4** and **Figure 5**)

Most importantly, the significant weight loss in patients did not lead to a simultaneous decrease in Fat-Free Mass (FFM) and indexed FFM (FFMI) (**Table 4**). As illustrated in **Figure 5**, a significant increase in Fat-Free Mass Index was observed as early as T3 compared with T0, and the increase, although no longer significant, was confirmed until the end of the follow-up. Concordantly, both Skeletal Muscle Mass (SMM) and Skeletal Mass Index (SMI) were found to be preserved during therapy, despite no specific exercise recommendations were provided; indeed, far from decreasing, they increased, although not significantly, throughout the observation period (**Table 4** and **Figure 5**). This resulted in a beneficial effect on the SMM/VAT ratio, a well-known index inversely related to CVR and sarcopenic visceral obesity (36). In fact, this ratio increased significantly at T3 compared with T0, and remained higher than at baseline at T6, although the latter change was no longer statistically significant (see **Figure 5**).

Finally, analysis of impedance data on body water distribution showed an improved overall hydration (significant increase in total body water) and more advantageous fluid distribution (no increase in ECW/TBW ratio), (**Table 4**).

### 4.5 Side effects

**Figure 6:**
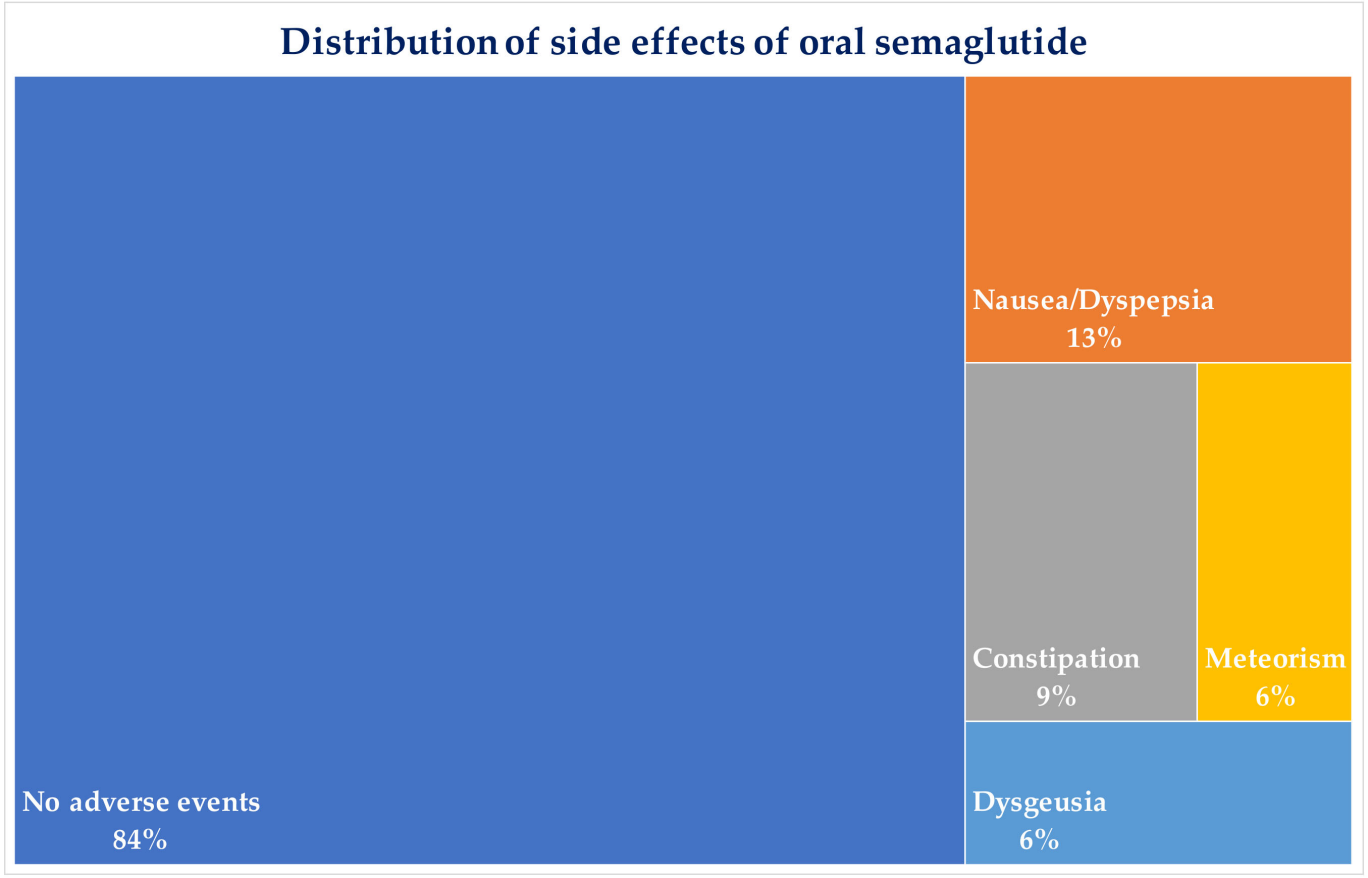
Distribution of major side effects complained by patients during treatment with oral semaglutide. Percentages are related to the total number of patients (n=32).

**Figure 7:**
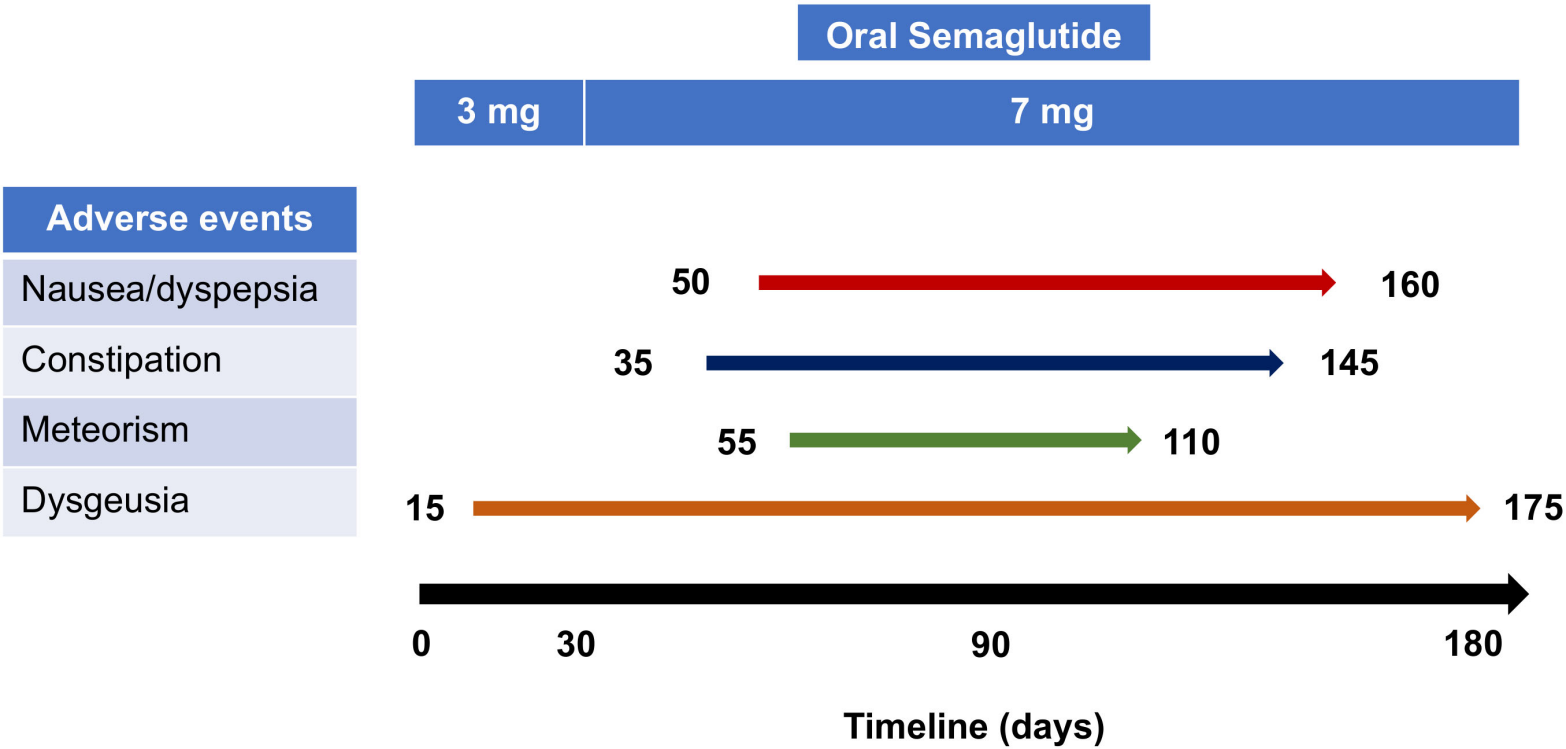
The graph highlights the occurrence of side effects during the observation period. Most of them occurred after at least one month of treatment, indicating that the dose adjustment of semaglutide (3 to 7 mg after 30 days of treatment) was the cause of these complaints. Meteorism was the most transient side effect, disappearing in 55 days. Dysgeusia is one of the latest classified side effects, as suggested by post-marketing data, and was recently included among the side effects of oral semaglutide by the European Medicines Agency. Dysgeusia was described early after two weeks of treatment (3 mg/day) and consisted of a metallic taste in the tongue and mouth, which occurred in only 6% of patients but persisted throughout observation.

Oral semaglutide was found to be well-tolerated, and no patient discontinued the treatment until the end of follow-up. The main side effects complained of by patients were generally mild and mostly transient, and their hierarchical distribution is shown in **Figure 6**. Patients who experienced side effects reported that these occurred mainly from the second month of treatment, and most of them soon after titration of semaglutide to 7 mg/day. **Figure 7** shows detailed information on the onset and evolution of side effects.

## 5 Discussion

The results of this 26-weeks prospective, real-life study show that the oral formulation of semaglutide, in addition to improving glucose control and body weight, exerts beneficial effects on body composition by reducing fat mass and preserving muscle mass, with better overall body fluid distribution.

Since 2005, GLP-1RAs have been a treatment option for T2D, providing a significant reduction in HbA1c and body weight with a low risk of hypoglycemia. They have also been shown to provide cardio- and nephron-protection, especially in patients with cardiovascular and chronic kidney disease (37–39). Currently, semaglutide is the first GLP-1RA designed for both oral and injective administration. The oral formulation is an appealing option for patients with T2D who prefer oral to injectable therapies. By increasing patient compliance and adherence, oral semaglutide could promote earlier use of GLP1-RAs, allowing them to act as disease modifiers. It also provides a viable alternative during the current period of international shortage of the subcutaneous formulation of semaglutide (40).

Our study population also included newly diagnosed patients (disease duration 0 years). The short median duration of T2D, less than 10 years, indicates full implementation of recent guidelines prescribing GLP1-RAs as one of the main classes to be used in the first instance. At the same time, patients with long-lasting disease (maximum duration 35 years) were not excluded from the study, suggesting that oral semaglutide may also be a therapeutic choice in patients with longer disease evolution. Normal BMI was not a barrier to prescribing oral semaglutide, as normal-weight patients were also treated, as previously suggested (6). It should be considered, in fact, that the link between CVR and BMI is complex and related not only to the amount of body weight, but rather to fat distribution and body composition. Thus, normal weight patients may have a CVR similar to that of overweight individuals if their fat mass is predominantly visceral and/or they have a small amount of muscle mass (sarcopenia).

The reduced oral bioavailability (0.4-1%) and complex pharmacokinetics of oral semaglutide make appropriate counseling and patient adherence to the administration instructions crucial, and are potential obstacles to the success of this treatment regimen. The absorption of oral semaglutide, in fact, largely depends on several factors, including a fasting state and no pharmacological interference. It should be taken fasting, swallowing the tablets with about 120 ml of water, and then fasting for at least another 30 minutes post-administration (41). Hence, the bioavailability could be affected leading to a larger patient-to-patient variability when compared to subcutaneously injectable semaglutide (42). In this context, our evaluation of the effects of a 26-week course of oral semaglutide therapy confirmed, in a real-life setting, the effectiveness of this new drug formulation in achieving an early and significant decrease in HbA1c, body weight and waist circumference in treated patients. With reference to the body weight parameter, the average body weight loss was about 4 kg after 6 months. The result agrees with data from the main PIONEER studies (10, 14), with a response rate of 50%, which should be considered a satisfactory endpoint in this population. Indeed, in enrolled patients who were normal or underweight, excessive body weight loss was not desirable. A good drug manageability emerged from its negligible effects on heart rate, eGFR, and pancreatic enzymes. Furthermore, the significant reduction in ALT and FLI suggests that oral semaglutide improves liver lipotoxicity due to NAFLD, as previously reported for injectable GLP1-RAs (43) and, recently, for oral semaglutide as well (44). The significant change in FLI and not HSI in our study population, which had no signs of NASH (normal liver enzyme levels) at baseline, would reflect the predominant effect of the drug more on waist circumference, which enters the calculation of FLI alone and is more related to visceral fat, than on liver enzymes. Finally, also the finding of unchanged values of APRI and FIB4 scores under semaglutide therapy was not surprising. Indeed, both scores are validated tools for delineating the risk and severity of liver fibrosis and cirrhosis even in patients with NAFLD. In our sample, median values were normal at baseline, indicating that the study population was at low risk for liver fibrosis. Therefore, the absence of deterioration of these clinical data was what was expected over such a short observation period.

Considering the significant effects of GLP-1RAs on body weight reduction, it has become essential to assess their impact on different body compartments, and especially metabolically active components. In people who are overweight or obese, the decrease in fat-free mass (FFM), which includes the total mass of bone, muscle, connective tissue, and fluids within the body, contributes about 20-30% of the total *diet-i*nduced weight loss (45). Strategies to preserve muscle mass and function in patients with T2D receiving a dietary or therapeutic regimen that may induce weight loss are therefore crucial in the management of the disease (46), especially considering that T2D patients are prone to sarcopenia (20). Studies focusing on GLP1-RA effects on body composition have been concordant in demonstrating the effectiveness of this drug class in reducing fat mass while preserving lean mass (26–28, 47). Few studies have yet addressed this issue with reference to oral semaglutide. A 12-week randomized controlled trial (RCT) on T2D patients recently described a reduction in food craving, preference for sweet foods, and improved dietary control with oral semaglutide compared with placebo, resulting in a decrease in body fat mass analyzed by plethysmography, without impairment of whole lean mass (48).

In our study, during therapy we observed a significant and early decrease in both total fat mass and the fat mass index (FMI), which latter is a more reliable parameter for inter-individual comparisons because it normalizes measurements to height. Visceral adipose tissue also decreased during follow-up, although the reduction was not statistically significant. This finding is not surprising considering the baseline characteristics of the enrolled patients, which initially had pathological but highly variable average values of visceral adipose tissue, with minimum values as low as 0.6 liters, within normal range. The loss of visceral adipose tissue, regardless of magnitude, still has beneficial effects on glucose metabolism and is accompanied by a decrease in circulating cytokines involved in low-grade inflammation, insulin sensitivity, and cardiovascular risk (49).

Undoubtedly, the most interesting data concern the impact of oral semaglutide on lean mass. Throughout the observation period, despite the significant weight loss, patients had a beneficial and significant increase in Fat-Free Mass as early as the third month of treatment that persisted until the end of follow-up. Notably, this effect was attributable in part to the preservation of skeletal muscle mass and improved distribution of body fluids. This is in line with studies showing that GLP-1 increases vascular blood flow and activates glucose delivery into skeletal muscle via AMP-activated protein kinase (AMPK), thereby promoting muscle synthesis, reducing muscle breakdown (50, 51) and improving muscle microvascular recruitment (MVR) through increased endothelial surface area (52). Skeletal muscle mass enhancement positively affects insulin sensibility and fat metabolism through different molecular mechanisms (53), ultimately leading to clinically relevant benefits.

The finding of higher values of the SMM/VAT ratio after therapy than at baseline, suggests that oral semaglutide could prevent a potential state of sarcopenic visceral obesity in T2D patients. This is an important therapeutic goal because the risk of sarcopenic obesity increases with aging and is associated with worse glucose and blood pressure control, higher insulin resistance, higher prevalence of dyslipidemia, T2D, cardiovascular diseases, NAFLD, and higher mortality than sarcopenia or obesity alone (54, 55). In addition, the condition characterized by poor skeletal muscle mass and visceral adipose tissue accumulation (the so-called “sarcopenic visceral obesity”), is associated with a further increased insulin resistance and impaired metabolism, worsening the overall clinical picture (55).

Finally, special attention should be paid to the analysis of impedance data on body water distribution. The ECW/TBW ratio is strongly indicative of the body nutritional and metabolic qualities (56). Our data suggest that patients had a better hydration, as indicated by significant increase in total body water, and more beneficial fluid distribution (no increase in the ECW/TBW ratio) at the end of follow-up, indicating an overall improvement in the body’s nutritional status as a result of therapy.

To our knowledge, this is the first prospective real-world study to evaluate the impact of oral semaglutide on body composition and metabolic parameters in overweight and normal-weight patients with T2D. The main limitations of the study are the lack of a control group, the single-center design, and the low sample size, which, although statistically adequate, does not allow for a more in-depth analysis of muscle mass stratified by patient gender. Another limitation is the lack of instrumental evaluation of the liver, which did not allow us to confirm the diagnosis of NAFLD and its related liver complications. Finally, in light of the short duration of follow-up, it should be pointed out that long-term studies are needed to evaluate the duration of the effects of oral semaglutide over time.

## 6 Conclusions

Our data confirm the effectiveness of oral semaglutide, showing early benefits on glucose control, body weight, and fat mass. Oral semaglutide was also found to be a useful option for approaching normal-weight T2D patients, who are often excluded from GLP1-RA treatment. Of particular interest were the unexpected effects on lean mass. Improvement of muscle mass is highly desirable in patients with diabetes, who are more prone to sarcopenia conditions. This field of action is increasingly emerging as an interesting challenge that opens new promising perspectives in the multiparametric and personalized management of diabetic disease.

## Data availability statement

The raw data supporting the conclusions of this article will be made available by the authors, without undue reservation.

## Ethics statement

The studies involving humans were approved by Ethics Committee of the Bari Polyclinic University Hospital. The studies were conducted in accordance with the local legislation and institutional requirements. The participants provided their written informed consent to participate in this study.

## Author contributions

GP, SV, and DR contributed to conception and design of the study. VC, VL, DT, LC, and GL organized the database. GP, GL, VC, and VL contributed to the clinical follow-up of patients. MF performed the statistical analysis. SV, GL, and GP wrote the first draft of the manuscript. GP, SV, GL, DR, VT, CS, and GDP contributed to review and editing the final version of the manuscript. All authors contributed to the article and approved the submitted version.
